# Point cloud registration from local feature correspondences—Evaluation on challenging datasets

**DOI:** 10.1371/journal.pone.0187943

**Published:** 2017-11-14

**Authors:** Tomas Petricek, Tomas Svoboda

**Affiliations:** Department of Cybernetics, Faculty of Electrical Engineering, Czech Technical University in Prague, Prague, Czech Republic; University of Texas at San Antonio, UNITED STATES

## Abstract

Registration of laser scans, or point clouds in general, is a crucial step of localization and mapping with mobile robots or in object modeling pipelines. A coarse alignment of the point clouds is generally needed before applying local methods such as the Iterative Closest Point (ICP) algorithm. We propose a feature-based approach to point cloud registration and evaluate the proposed method and its individual components on challenging real-world datasets. For a moderate overlap between the laser scans, the method provides a superior registration accuracy compared to state-of-the-art methods including Generalized ICP, 3D Normal-Distribution Transform, Fast Point-Feature Histograms, and 4-Points Congruent Sets. Compared to the surface normals, the points as the underlying features yield higher performance in both keypoint detection and establishing local reference frames. Moreover, sign disambiguation of the basis vectors proves to be an important aspect in creating repeatable local reference frames. A novel method for sign disambiguation is proposed which yields highly repeatable reference frames.

## Introduction

Point cloud registration has many applications including mobile robotics, object modeling, and object recognition and pose estimation. It is a crucial step of the most commonly used methods for Simultaneous Localization and Mapping (SLAM), whether operating on the data from laser scanners or consumer-electronics RGB-D sensors, which have become widely available.

A variant of the Iterative Closest Points (ICP) algorithm is often employed to solve the task— see [1, 2] for the seminal papers on its point-to-point and point-to-plane formulations, respectively, or [3] for a generalization of these two methods. Despite many advantages of the algorithm, including real-time operation in some settings, the ICP algorithm has several drawbacks. Being an iterative local minimization method, it is sensitive to the initial alignment of the point clouds to be registered and their mutual overlap. As shown by [4], an inaccurate initial alignment or a low overlap between laser scans may deteriorate the accuracy of registration severely.

In order to overcome the limitations of the ICP algorithm, methods to establish global correspondences based local feature descriptors were suggested, such as [5, 6]. Since ICP performs very well if started within the basin of convergence, the coarse alignment obtained from these global methods often serves as an initial guess for ICP [6]. In modeling of objects from their partial views, ICP has been used to verify established correspondences and to refine registration provided from these [7].

Several alternative approaches have also been proposed. Instead of the special-purpose ICP formulation, Fitzgibbon [8] approached the registration problem as a general non-linear optimization which allowed to incorporate robust estimation via a Huber kernel. In 3D Normal-Distribution Transform (3D-NDT) [9], the surface is represented by a Gaussian Mixture Model and registration is also carried out by standard methods from numerical optimization. 4-points congruent sets (4PCS) are sought and matched in [10]. Despite the fast matching procedure proposed in the paper, for *n* input points the number of all possible coplanar 4-tuples is still $O(n^{4})$, which presents a major issue, especially for scans with large planar regions. The computational efficiency of this method was later addressed in [11] by creating 4-tuples from sparse local features instead of points.

**Fig 1:**
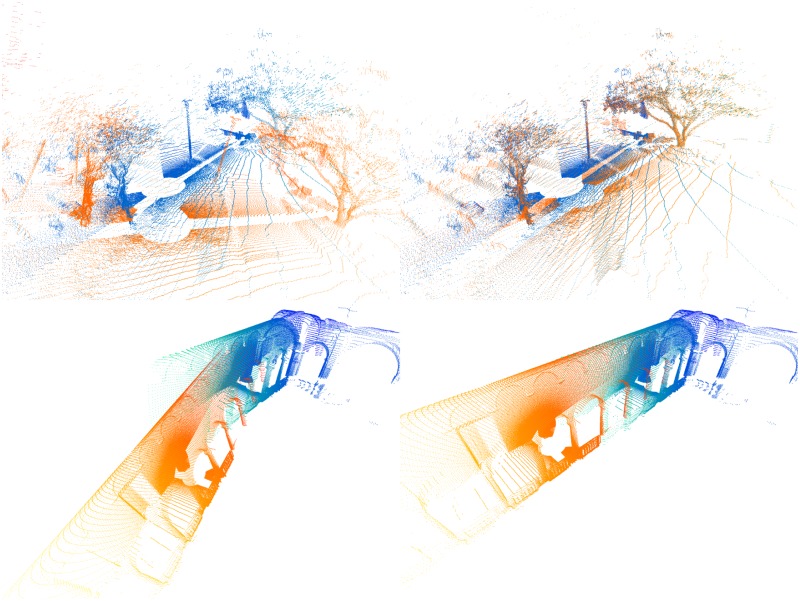
Data from the experimental protocol. Point clouds from two datasets—(top) Gazebo with overlap 0.9 and (bottom) ETH with overlap 0.59. Reading and reference point clouds (left) prior registration and (right) aligned according to ground truth. The reference is displayed in blue, the reading in orange tones.

Even though many registration algorithms have been proposed, their fair comparison is still difficult due to a lack of datasets which would capture variety of scenes robots may encounter in the real world. A notable contribution to this area is due to [4, 12] which provide an experimental protocol using six medium-sized datasets with accurate ground-truth poses, capturing diverse environments, both indoor and outdoor, ranging from an apartment to a woodland area. This experimental protocol constitutes a basis for our evaluation. An example pair of reading and reference point clouds are shown in Fig 1. The same protocol has previously been used in evaluating 3D-NDT in [13, 14].

The contribution of the paper is threefold.

- We extend the local features from [15] by introducing keypoint detection and modifying the underlying method for establishing local reference frames. The method is evaluated on challenging real-world datasets, showing that for a moderate overlap between the laser scans, it provides a superior registration accuracy compared to four local methods [1–3, 9] and another three global methods [5, 10, 11].
- Underlying components of the method, namely the keypoint detection and the local reference frames, are evaluated with respect to the task, along with the effects of their respective parameters, and general suggestions are given concerning specific design choices.
- For local reference frames, we compare three methods for sign disambiguation of the basis vectors. One of these methods is novel and achieves better repeatability than the general method of [16] used in the Signature of Histograms of Orientations (SHOT) [17]. The results also justifies using the sensor position for sign disambiguation in situations when it is known.

## Methods

### Feature-based registration

We formulate the registration task according to [4]. Given two point clouds, *reading* $P_{1}\subset R^{3}$ and *reference* $P_{0}\subset R^{3}$, the task is to find a rigid transformation **T**_0←1_ such that **p**_0_ = **T**_0←1_(**p**_1_) for corresponding points $p_{1}\in P_{1}$, $p_{0}\in P_{0}$. In homogeneous coordinates, this is a linear transformation

$$\begin{array}{c} {}[\begin{array}{c} p_{0}\\ 1 \end{array}]=T_{0\leftarrow 1}[\begin{array}{c} p_{1}\\ 1 \end{array}]=[\begin{array}{cc} R_{0\leftarrow 1} & t_{0\leftarrow 1}\\ 0^{⊤} & 1 \end{array}][\begin{array}{c} p_{1}\\ 1 \end{array}], \end{array}$$ (1)

with **R**_0←1_ being a 3-by-3 rotation matrix, **t**_0←1_ a 3-by-1 translation vector, and **0**^⊤^ a 1-by-3 zero vector. Points **p**_*i*_ can be assigned additional properties, such as surface normal **n**_*i*_, saliency *h*_*i*_, or a descriptor **d**_*i*_, where the subscript denotes the index of the corresponding point.

The task is directly related to finding correspondences from the reading to the reference. From a set of tentative correspondences, found by matching local descriptors of the data, the transformation can be estimated using a robust estimator, such as Random Sample Consensus (RANSAC) [18].

We first introduce a general framework for feature-based registration. Within such a framework, underlaying components of the method are then evaluated.

### Keypoint detection

Keypoints are selected as extrema of a saliency measure, which determines the kind of structures being sought in the data and directly affects repeatability and robustness of the detection. Fixed-scale and adaptive-scale detectors can be distinguished [19]—the former are given scale as a parameter, the latter seek characteristic scales within a scale-space representation of the data, which need to be constructed for these purposes. We will not consider scale-adaptive detectors for the registration task since the scale is not ambiguous with the data from calibrated sensors, relevant scale changes are unlikely to occur in reality, and because seeking characteristic scale introduces an additional source of errors which affect all the following stages. We restrict feature matching to include only features of the same scale.

Local extrema are obtained via non-maxima suppression where only the keypoints with locally maximal saliency are retained. Specifically, a keypoint at point **p** with saliency *h* is kept only if *h* ≥ *h*_*i*_ for all $i\in N_{\sigma }\left(p\right)$, where $N_{\sigma }\left(p\right)$ is a set of point indices within the *σ*-neighborhood of **p**. Points **p** with $|N_{\sigma }\left(p\right)|<10$ are excluded from keypoint detection.

We consider two types of keypoint detectors. The first uses the covariance matrix of points,

$$\begin{array}{c} C_{p}=\frac{1}{\sum_{i}w_{i}}\sum_{i\in N_{s}\left(p\right)}w_{i}\left(p_{i}-\mu \right){\left(p_{i}-\mu \right)}^{⊤}, \end{array}$$ (2)

where $\mu =\frac{1}{\sum_{i}w_{i}}\sum_{i}w_{i}p_{i}$, the second type uses the covariance matrix of normals,

$$\begin{array}{c} C_{n}=\frac{1}{\sum_{i}w_{i}}\sum_{i\in N_{s}\left(p\right)}w_{i}n_{i}n_{i}^{⊤}, \end{array}$$ (3)

where *w*_*i*_ are weights assigned to individual points, $N_{s}\left(p\right)$ is a set of neighboring points of **p**, {*i* ∣ ‖**p**_*i*_ − **p**‖ ≤ *s*}.

The eigenvalues of these covariance matrices will be denoted by λ_1_, λ_2_, λ_3_ in their decreasing order, and their corresponding eigenvectors by **q**_1_, **q**_2_, **q**_3_. We consider the following saliency measures as functions of the eigenvalues: $\min(\frac{\lambda_{1}}{\lambda_{2}},\frac{\lambda_{2}}{\lambda_{3}})$, λ_1_, λ_2_, λ_3_, $\frac{\lambda_{1}}{\lambda_{2}}$, $\frac{\lambda_{2}}{\lambda_{3}}$.

Despite an intuitive geometrical meaning of the saliency measures, this may not directly correspond to their quality in terms of repeatability of the corresponding keypoints. Therefore, we evaluate several such measures in order to select the most suitable for the task at hand.

Some of these saliency measures have previously been used. For example, [20] uses the smallest eigenvalue λ_3_ of **C**_p_ and several methods based on **C**_n_ have been implemented in the Point Cloud Library [21], including the one using λ_3_, which can be seen as a direct extension of [22] but replacing the image gradients with the surface normals. We provide an experimental evaluation which compares them with possible alternatives and justifies their usage with challenging real-world data.

For the keypoints found in this stage we establish local reference frames and compute the descriptors.

### Local reference frames

Local reference frames are the key means to achieve the desired level of descriptor invariance. Although the Cartesian coordinate system is the most common today [15, 17, 20], there are methods using a single reference axis [23], or no local frames at all [5]. Using reference frames yields several advantages. First, the three-dimensional distribution of points can be captured by the descriptor to increase its discriminative power. Second, each feature correspondence can provide an estimate of the transformation between the laser scans.

As noted by [17], although many methods rely on repeatable local frames, the importance of its particular choice is underrated. A common approach followed by many methods is to establish the basis of the reference frame from the eigenvectors of the feature covariance matrices as defined above.

As discussed in [16], singular value decomposition (SVD) of a matrix is unique only up to a reflection of each pair of singular vectors **u**_*i*_,**v**_*i*_ since $\sigma_{i}u_{i}v_{i}^{⊤}=\sigma_{i}\left(-u_{i}\right){\left(-v_{i}\right)}^{⊤}$ for every pair of singular vectors. The same applies to eigenvalue decomposition of real symmetric matrices. Disambiguating the sign of the eigenvectors is thus needed to obtain a unique and unambiguous reference frame [17]. Right-handedness of the reference frame is then enforced by setting one of the basis vectors to the cross product of the remaining two.

Zhong [20] uses the eigenvectors of the point covariance matrix **C**_*p*_ but does not disambiguate their signs. Tombari *et al*. [17] use the eigenvectors of the point covariance matrix **C**_*p*_, replacing ***μ*** by the feature position **p** and using *w*_*i*_ = 1 − ‖**p**_*i*_ − **p**‖/*s* for the weights, *s* being the scale, and follow the general procedure of [16] to disambiguate signs. The eigenvectors of **C**_*n*_ are used in [15], with weights *w*_*i*_ assigned based on the surface area of the respective polygons.

Throughout this paper, **Q**_*i*_ denotes the orthonormal basis of the local reference frame associated with point **p**_*i*_, and contains the eigenvectors **q**_1_, **q**_2_, **q**_3_ in its columns. We consider three different methods of sign disambiguation, applied individually to each eigenvector **q**.

- The first, used in [15, 17], changes the sign of **q** to make ∑_*i*_ sign(**p**_*i*_ − **p**)^⊤^ **q** positive—we refer to this method as *support*.
- The second, denoted *mean*, reverses the sign of **q** if (*μ* − **p**)^⊤^ **q** < 0, where **p** is the feature position and *μ* the centroid of the points within the local neighborhood defined above. This method has not been used, to our knowledge, to establish local reference frames.
- The third, denoted *sensor*, assumes the sensor origin **s** is known and reverses the sign if (**s** − **p**)^⊤^ **q** < 0. This is a commonly used method for ensuring consistent orientation of estimated surface normals when the sensor position is known, yet it is less common in disambiguating all axes of local reference frames.

### Feature descriptor

**Fig 2:**
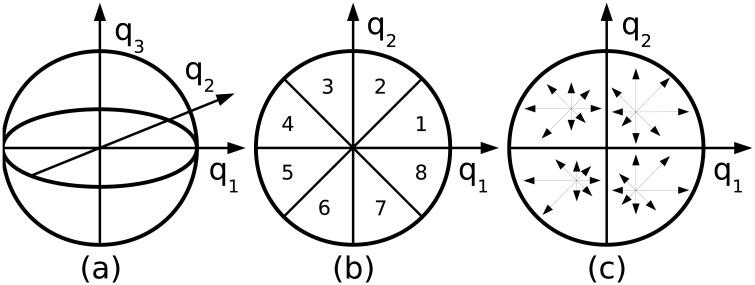
Feature descriptor. (a) spherical support with local reference frame, (b) 8 orientation bins, (c) 4 spatial bins.

We use the descriptor of [15] with 3 × 3 in-plane spatial bins and 8 polar bins. The descriptor is created by projecting the points within the neighborhood and their corresponding normals onto three planes spanned by pairs of the basis vectors, and accumulating the projections into histograms. Each oriented point casts weighted votes into the two nearest polar bins, given by the normal projection, and into the four nearest spatial bins, given by the point projection. The weights are proportional to relative proximity to each histogram bin and inversely proportional to the local surface sampling density (the area of the corresponding polygon was used in [15]). See Fig 2 for an illustration, and [15] for more details regarding the descriptor and its application to object recognition.

### Pose from correspondences

Invariant descriptors are used to establish tentative correspondences from reading to reference. Let **p**_1_, **p**_0_ be a pair of points from reading and reference, respectively, and **d**_1_, **d**_0_ their associated descriptors. Then a correspondence is established if **d**_0_ is the among three nearest neighbors of **d**_1_ from the set of the reference descriptors and **d**_1_ is among three nearest neighbors of **d**_0_ from the set of the reading descriptors.

The pose is estimated from the set of tentative correspondences via Locally Optimized RANSAC [24]—pairs of correspondences, drawn randomly from the set, generate model poses and the pose maximizing the number of consensual correspondences is sought. The maximum number of iterations is estimated online, by setting the probability of missing the inlier set to *η* = 1/100 (see [24] for details). To generate the model poses, and to refine the pose from consensual correspondences, we use the method of [25] to find

$$\begin{array}{c} \underset{R_{0\leftarrow 1},t_{0\leftarrow 1}}{\mathrm{argmin}}\sum_{i}\|R_{0\leftarrow 1}p_{1,i}+t_{0\leftarrow 1}-p_{0,i}{\|}^{2}+{\left\|R_{0\leftarrow 1}n_{1,i}-n_{0,i}\right\|}^{2} \end{array}$$ (4)

for matching point positions **p**_0,*i*_, **p**_1,*i*_ and normal vectors **n**_0,*i*_, **n**_1,*i*_. Before creating the model pose, we check that feature distances are consistent among both the point clouds and ignore the inconsistent samples.

Correspondences of the surface normals were used to generate model poses (from a pair of correspondences) and to locally optimize the model when up to five consensual correspondences were available. With more correspondences, the benefit of these terms vanished and minimizing the criterion based solely on the corresponding positions yielded more accurate pose estimates. Inlier threshold for a correspondence to be considered consensual was twice the scale of non-maxima suppression.

### Dataset and experimental protocol

The laser registration datasets of [12] are used for experimental evaluation of the method and its components. The datasets were recorded with a laser rangefinder mounted on a tilting platform. For each scan the ground-truth position and orientation was obtained using a theodolite. The datasets contain both indoor and outdoor scenes, structured (an apartment, buildings) and unstructured environment (woodland area, a mountain plain), and dynamic elements with varying time spans (intra-scan and inter-scan motions, seasonal changes).

An experimental protocol for evaluation of point cloud registration methods is introduced in [4] by selecting pairs of scans from the datasets. From each dataset, 35 pairs of laser scans were selected to ensure approximately uniform coverage of the scan overlap from 0.3 to 0.99. The overlap is defined as the ratio of points from $P_{1}$ for which a matching point exists in $P_{0}$.

For each pair of point clouds 3 × 64 perturbations from the ground-truth alignment were generated to serve as initial alignments, 64 from each of the three Gaussian distributions with increasing variance. This establishes three classes of registration tasks with increasing difficulty, called *easy*, *medium*, and *hard* poses and constitutes a common ground for assessing sensitivity to initial alignment. As the feature-based methods are mostly insensitive to initial alignment of the point clouds we only use the first *hard* pose for each pair for their evaluation.

After transforming the reading point cloud to the initial pose, both point clouds are preprocessed as follows. First, the points with distance to the sensor less than 1m or greater than 20m are removed. Then, the point clouds are subsampled to achieve a maximum sampling density about 100m^−2^ and surface normal is estimated at each point kept by fitting a plane to its 15 nearest neighbors before subsampling. The normals are reoriented to point towards the sensor.

The datasets are particularly challenging due to several reasons. Our results suggest that the main difficulty comes with a low overlap between some of the point cloud pairs and sometimes prevailing repetitive structures, especially in the *ETH* dataset. Variations due to viewpoint change, sampling and noise seem to be relatively high compared to those induced by the variability in the scene itself.

Another difficulties comes with the sensing device—large parts of the scene are occluded by the moving platform itself, namely the poles on which the prisms are mounted. Tilting the laser also causes a very nonuniform sampling density, which increases towards the axis of rotation. Nevertheless, these are all difficulties which might need to addressed in applications and therefore we consider this to be a good benchmark for evaluation of registration methods.

## Results

Prior to evaluating the registration method as a whole following the protocol of [4], we evaluate keypoint detection and local reference frames using a small number of laser scans and fix their parameters. The following parameter choices are assessed:

- type of the features used (points, normals),
- scale of the keypoint detection and the local reference frames,
- weights assigned to the features (normalized distance from feature point [17], surface area [15]),
- method of sign disambiguation (support, mean, sensor),
- pairs of basis vectors to ensure right-handedness of the local reference frames (**q**_1_ × **q**_2_, **q**_2_ × **q**_3_, **q**_3_ × **q**_1_).

### Repeatability of keypoint detection

For keypoint detection we measure relative keypoint repeatability similarly to [19], as the ratio of the repeatable keypoints to all keypoints extracted from the reading. A keypoint is said to be repeatable if, after being transformed into the reference by the ground-truth transformation **T**_0←1_, its nearest neighbor among the keypoints detected in the reference is closer than some threshold. We set this threshold to be the same as the scale of non-maxima suppression.

As discussed in [26], the density of the extracted keypoints may affect the repeatability score—trivially extracting all the points would yield high repeatability. Thus, we include an experiment similar to the “Quantity Bias” experiment from [19], where only a limited number of the most salient keypoints are extracted to evaluate the keypoint detector. The repeatability score is also computed for the same number of randomly extracted points to assess the ratio of keypoints being matched by accident.

Regarding the feature weights, we have not found any to be significantly better than the others across all scales, both in keypoint detection and local reference frames, and therefore only the unit weights are considered further. From scales ranging from 0.25m to 1.0m, 0.35m provides best performing parameter combinations and is therefore selected to report the quantitative results below. This scale is also used in the point cloud registration experiments.

**Fig 3:**
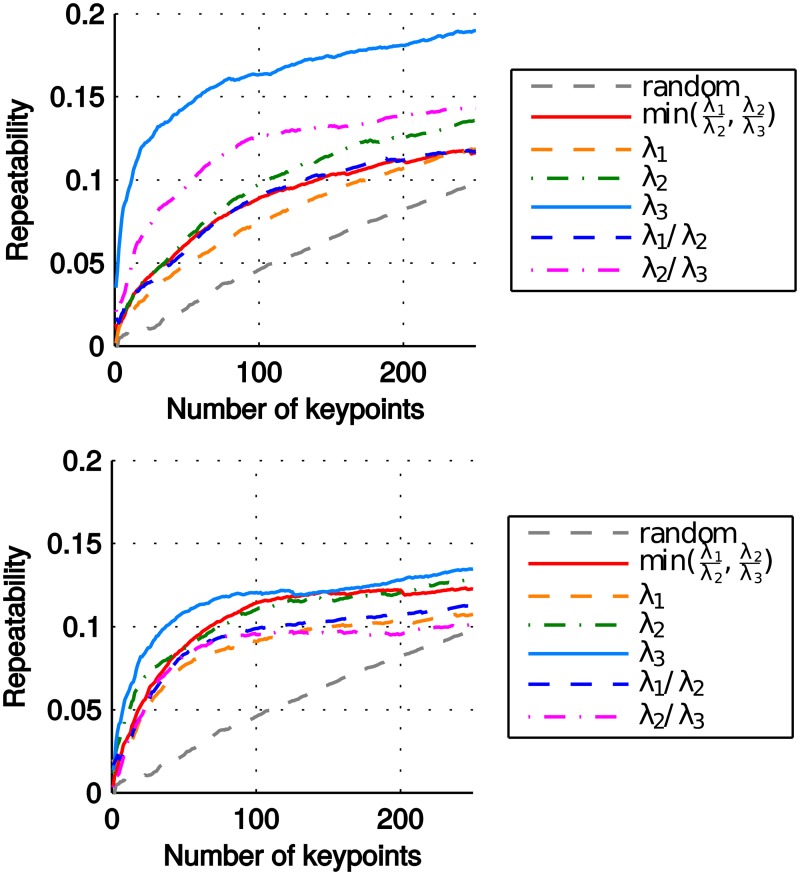
Repeatability of keypoints. Repeatability of keypoints from (top) points and (bottom) normals for each saliency measure.

For each feature type, we report the results for the saliency measures in Fig 3. The saliency measure λ_3_ provides the best results for both feature types, with a large margin for points. It selects the regions where the minimum variance of the features in any direction is locally maximum, informally speaking, where the features spread in all directions most evenly. Note that the relative order of the saliency measures tends to be stable with the increasing number of selected keypoints, with only a few exceptions.

### Repeatability of local reference frames

To evaluate the repeatability of the local reference frames, several metrics have been proposed— [17] measures the mean cosine of the corresponding axes, [27] aligns the *z* axes before measuring the cosine of the *x* axes to decorrelate the two measurements. In our experiments, we apply the same metric we use to quantify the rotation error of the registration itself.

Specifically, if **Q**_1_ and **Q**_0_ are the bases of the corresponding local reference frames from the reading and the reference, respectively, and **R**_0←1_ is the ground-truth rotation, the displacement of the reference frames is computed as

$$\begin{array}{c} e_{q}=\arccos(\frac{\text{tr}(R_{0\leftarrow 1}Q_{1}Q_{0}^{⊤})-1}{2}). \end{array}$$ (5)

This measures the minimum angle of rotation needed to align the two bases and provides an upper bound on displacements of individual axes.

For the selected pairs of laser scans, 250 points are randomly selected from their overlapping parts where the local reference frames are established. The displacement *e*_q_ is then computed for such corresponding reference frames.

As mentioned above, we further consider only the unit weights as other alternatives do not provide significant advantage. From scales 0.5m to 2.0m, the larger ones were found to provide more repeatable local reference frames and were also most frequent among the best performing combinations. All the results below are given for the scale fixed to 2m.

**Fig 4:**
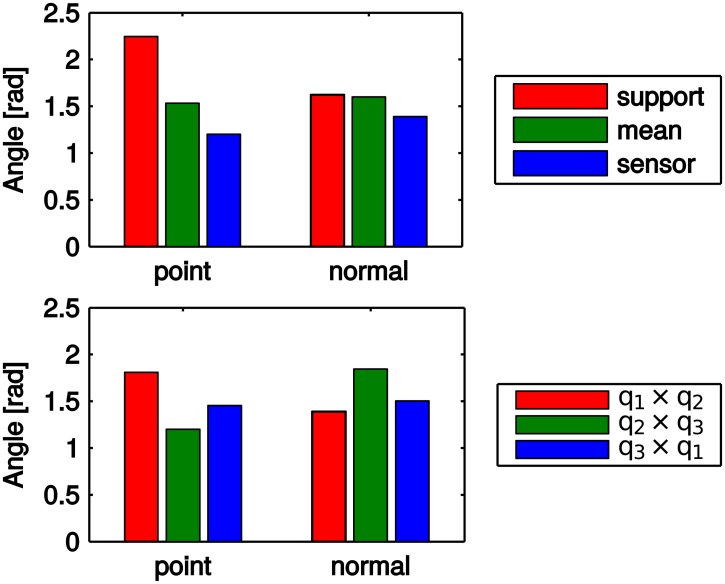
Local frame displacement. Average displacement *e*_*q*_ of the corresponding local reference frames for (top) sign disambiguation methods and (bottom) pairs of disambiguated vectors ensuring right-handedness of the basis.

The average displacements for the sign disambiguation methods and the pairs of disambiguated vectors are shown in Fig 4. Note that we show the results with the remaining parameters having their optimal values—for example, the result for points and sign disambiguation based on the sensor uses **q**_2_ × **q**_3_ to comply with the right-hand rule but the similar result for normals uses **q**_1_ × **q**_2_ as these eigenvectors are easier to disambiguate for this feature type.

Fig 4 (top) shows that the sensor origin provides the strongest hint for the sign for both feature types, for points with a large margin, and therefore should be preferred to the others. In situations where the sensor origin is not available, the *mean* method outperforms the general method of [16, 17], here denoted *support*, when using points as features. For normals, these two methods perform comparably.

Fig 4 (bottom) shows that the most repeatable direction, including the sign, corresponds to the surface normal or a related direction, i.e., the 3rd basis vector for points, and the 1st basis vector for normals. The sign of this direction should therefore always be disambiguated directly, using an appropriate method, and not be given by the cross product of the remaining vectors.

### Registration

In this section, we evaluate our method using the experimental protocol described above and compare it to state-of-the-art methods [3, 5, 9–11].

Let ${\hat{T}}_{0\leftarrow 1}$ be the estimate of the ground-truth transformation **T**_0←1_ which aligns the reading with the reference. To assess the quality of the registration, [4] defines the residual transformation

$$\begin{array}{c} \Delta T={\hat{T}}_{0\leftarrow 1}T_{0\leftarrow 1}^{-1}={\hat{T}}_{0\leftarrow 1}T_{1\leftarrow 0}=[\begin{array}{cc} \Delta R & \Delta t\\ 0^{⊤} & 1 \end{array}] \end{array}$$ (6)

and computes registration errors *e*_r_ and *e*_t_ from its rotational and translational components, Δ**R** and Δ**t**, such that

$$e_{\text{r}}=\text{arccos}\left(\frac{\text{tr}\left(\Delta R\right)-1}{2}\right),$$ (7)

$$e_{\text{t}}=\left\|\Delta t\right\|,$$ (8)

where tr(Δ**R**) denotes the trace of Δ**R**. The rotation error *e*_r_ corresponds to the angle of rotation in the axis-angle representation. To allow an interpretation in terms of accuracy and precision, [4] suggests to use robust error statistics, namely the 50th, 75th and 95th percentiles of empirical distributions of the errors, referred to as A50, A75, A95. We follow this convention in our evaluation.

From local methods, we include in comparison Generalized ICP (G-ICP) [3] and 3D Normal-Distribution Transform (3D-NDT) [9] which is applied in a coarse-to-fine fashion with voxel sizes 2m, 1m and 0.5m. For a complementary evaluation of 3D-NDT, please refer to [13, 14, 28, 29]. From global methods, we include another method based on matching local features, namely the Fast Point-Feature Histograms (FPFH) with Sample Consensus Initial Alignment (SAC-IA) [5], and two alternative approaches, namely 4-Points Congruent Sets (4PCS) [10] and its keypoint-based variant (K-4PCS) [11].

In general, same preprocessing steps were used as for the proposed method except for 4PCS and K-4PCS for which we had to limit the number of input points to reduce running time, by using maximum density of 4m^−2^ and limiting maximum number of points to 500. For computing FPFH we used the common feature scale 2m, SAC-IA used three tentative correspondences for each feature and minimum feature distance of 1m to generate model poses. All the state-of-the-art methods were implemented in the Point Cloud Library [21].

**Fig 5:**
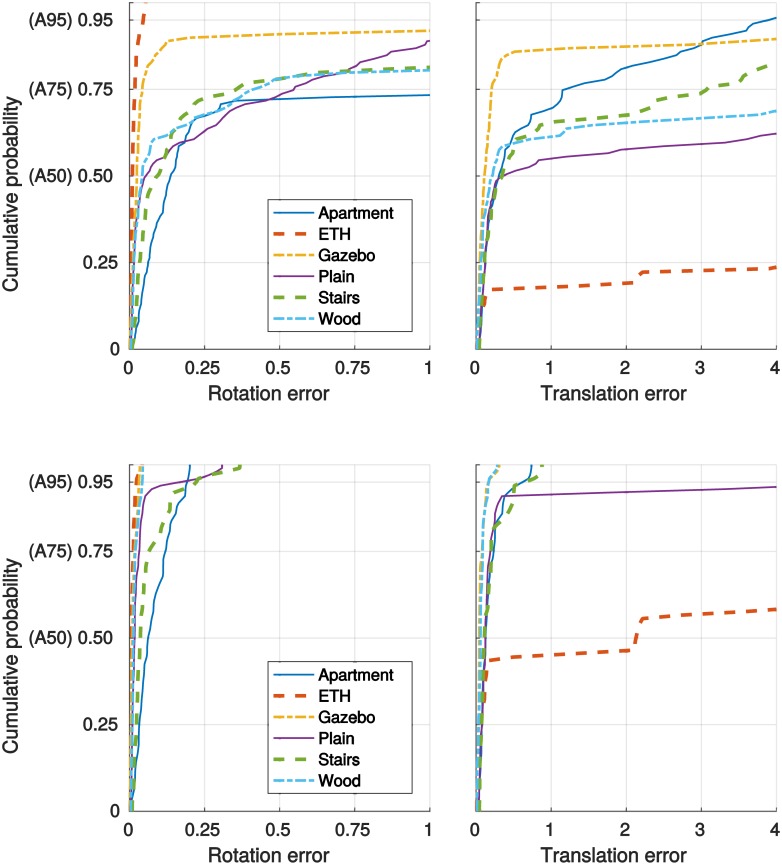
Point cloud registration accuracy. Distribution of (left) rotation and (right) translation errors for (top) all reading-reference pairs from hard poses and for (bottom) the pairs with overlap at least 0.75. A50, A75, and A95 denote the 50th, 75th, and 95th percentiles.

The rotation and translation errors, *e*_*r*_ and *e*_*t*_, for the samples from the *hard* poses are summarized in Table 1 (the best result for given percentile is typeset in bold). We also include the baseline results from [4] for the point-to-point (Point) [1] and point-to-plane (Plane) [2] ICP variants to allow easy comparison. Moreover, Fig 5 shows the full distributions of the errors achieved by the proposed method.

**Table 1:** Quantile statistics of registration errors.

		Apartment			ETH			Gazebo			Plain			Stairs			Wood		
		A50	A75	A95	A50	A75	A95	A50	A75	A95	A50	A75	A95	A50	A75	A95	A50	A75	A95
Rotation hard poses	Point [1, 4]	1.04	1.60	**2.53**	0.97	1.73	3.05	0.58	1.20	2.59	0.46	0.99	2.09	1.10	1.64	2.53	0.97	1.44	2.35
	Plane [2, 4]	1.01	1.72	2.95	1.31	2.09	3.11	0.58	1.31	2.88	0.50	1.09	3.05	1.48	1.91	2.94	1.05	1.56	2.53
	G-ICP [3]	0.73	1.74	3.10	0.74	1.43	3.03	0.38	1.13	2.92	0.07	0.86	2.97	0.56	1.63	3.07	0.57	1.26	2.72
	3D-NDT [9]	0.98	1.72	3.08	0.51	1.49	2.55	**0.02**	1.27	2.54	0.26	1.38	3.10	0.14	1.60	3.01	0.59	1.43	2.58
	FPFH [5]	0.51	1.96	3.13	0.03	0.05	0.64	0.13	0.30	1.38	0.16	**0.51**	**0.95**	0.44	2.31	2.99	0.28	**0.61**	2.42
	4PCS [10]	1.67	2.89	3.09	0.06	0.08	0.14	0.10	0.15	**1.07**	0.21	1.66	3.04	0.40	2.52	3.06	0.30	2.23	3.00
	K-4PCS [11]	1.81	2.94	3.12	0.13	0.35	3.03	0.40	2.17	2.98	0.29	0.77	2.69	0.99	2.68	3.05	2.09	2.81	3.10
	Ours	**0.14**	**0.35**	2.81	**0.01**	**0.02**	**0.04**	0.02	**0.04**	1.70	**0.05**	0.66	1.35	**0.10**	**0.23**	**1.83**	**0.04**	0.61	**2.28**
Translation hard poses	Point [1, 4]	1.29	1.99	**3.24**	3.84	7.06	14.77	1.58	2.79	**4.57**	2.02	3.14	**6.33**	1.81	2.78	4.75	2.32	**3.73**	**6.82**
	Plane [2, 4]	1.35	2.18	3.66	4.18	8.55	19.56	1.87	3.33	6.95	2.35	4.13	8.85	2.05	3.28	5.50	2.79	4.52	7.86
	G-ICP [3]	1.23	2.32	3.73	3.66	7.17	16.94	1.79	4.57	7.39	1.20	**3.09**	6.37	1.58	3.30	6.97	2.59	4.70	8.82
	3D-NDT [9]	1.24	2.22	3.58	**2.37**	**4.24**	**7.38**	**0.09**	2.76	5.02	2.02	3.69	7.01	1.30	2.62	**4.74**	1.72	4.15	7.54
	FPFH [5]	0.67	2.33	4.25	4.61	8.60	14.15	0.96	1.87	7.06	2.54	6.25	8.20	1.65	5.55	8.19	2.66	6.10	8.32
	4PCS [10]	1.91	3.03	4.62	4.43	9.74	14.27	0.79	1.31	5.88	2.86	9.06	15.37	1.49	5.63	10.08	2.57	6.78	10.09
	K-4PCS [11]	2.10	3.02	4.94	7.08	9.75	14.72	3.52	6.69	10.47	2.25	6.16	12.24	1.89	6.70	10.57	6.66	10.06	12.26
	Ours	**0.30**	**1.12**	4.04	6.33	10.63	14.42	0.13	**0.20**	5.21	**0.46**	6.79	9.37	**0.38**	**2.56**	7.49	**0.19**	6.20	10.53
Rotation hard poses overlap ≥ 0.75	Point [1, 4]	0.82	1.55	2.50	0.37	1.54	3.05	0.36	1.10	2.58	0.40	0.87	1.99	0.98	1.62	2.60	0.83	1.36	2.21
	Plane [2, 4]	0.81	1.64	3.00	0.02	1.85	3.12	0.40	1.16	2.70	0.42	0.93	3.05	1.40	1.82	2.94	0.89	1.46	2.46
	G-ICP [3]	0.16	1.63	3.10	0.60	1.24	3.06	0.02	1.01	2.95	0.01	0.72	2.90	0.02	1.57	3.08	0.01	1.18	2.74
	3D-NDT [9]	**0.04**	1.70	3.09	**0.01**	1.45	2.50	0.01	1.16	2.28	**0.01**	1.18	3.12	**0.01**	1.55	3.03	**0.01**	1.24	2.60
	FPFH [5]	0.11	0.30	1.68	0.02	0.05	0.06	0.03	0.06	0.12	0.04	0.07	0.48	0.06	0.10	2.91	0.13	0.16	1.16
	4PCS [10]	0.52	1.81	2.95	0.06	0.07	0.13	0.08	0.10	0.35	0.07	0.21	0.92	0.20	1.76	2.67	0.19	0.23	2.90
	K-4PCS [11]	0.43	2.91	3.10	0.08	0.28	3.04	0.07	0.17	2.16	0.15	0.31	2.08	0.34	2.73	3.04	0.39	2.98	3.12
	Ours	0.06	**0.12**	**0.20**	0.01	**0.01**	**0.02**	**0.01**	**0.02**	**0.03**	0.01	**0.04**	**0.08**	0.04	**0.08**	**0.21**	0.01	**0.02**	**0.04**
Translation hard poses overlap ≥ 0.75	Point [1, 4]	0.86	1.57	2.39	1.95	**3.30**	7.00	0.98	1.90	3.48	1.29	1.88	**3.06**	1.30	2.18	3.55	1.45	2.32	3.52
	Plane [2, 4]	0.83	1.70	2.68	2.19	4.30	9.54	1.23	2.70	5.87	1.40	2.40	4.95	1.42	2.47	4.08	1.86	2.84	4.60
	G-ICP [3]	0.41	1.72	2.83	2.39	5.51	13.70	0.06	3.47	6.50	0.07	1.58	4.22	0.06	2.42	6.53	0.09	3.02	7.83
	3D-NDT [9]	**0.06**	1.69	3.09	2.08	3.70	**5.38**	**0.04**	2.17	4.82	**0.05**	2.46	5.46	**0.04**	1.99	3.50	**0.03**	2.12	4.74
	FPFH [5]	0.27	0.49	2.18	**0.43**	4.47	7.32	0.26	0.37	0.75	0.39	0.81	3.52	0.22	0.50	7.86	0.36	0.81	2.22
	4PCS [10]	0.94	2.34	3.41	0.88	3.43	7.46	0.70	0.90	1.58	0.93	2.45	7.50	0.84	2.09	8.82	1.25	1.52	8.58
	K-4PCS [11]	0.69	1.57	4.90	4.56	5.28	8.11	1.02	2.18	5.48	1.33	1.85	5.30	1.34	5.53	10.57	1.93	8.21	11.42
	Ours	0.15	**0.25**	**0.68**	1.16	4.31	6.33	0.04	**0.12**	**0.29**	0.10	**0.21**	3.74	0.12	**0.20**	**0.76**	0.05	**0.10**	**0.27**

The table lists the 50th, 75th, and 95th percentiles (denoted A50, A75, and A95 respectively) of the rotation error *e*_r_ (in radians) and the translation error *e*_t_ (in meters). The two bottom blocks are restricted to the point cloud pairs with relative overlap at least 0.75. The results of the ICP variants [1, 2] are due to [4].

All methods considered in this paper fail in many cases, sometimes producing pose estimates which are further from the ground truth than the initial poses. Such results would most likely be unsatisfactory for any SLAM application. We also list the results for the reading-reference pairs with the overlap ratio at least 0.75 as the insufficient overlap seems to be the main cause why the methods based on matching invariant features are failing—see the bottom half of Table 1. Across higher overlap ratios, these methods yield good results in majority of cases.

**Fig 6:**
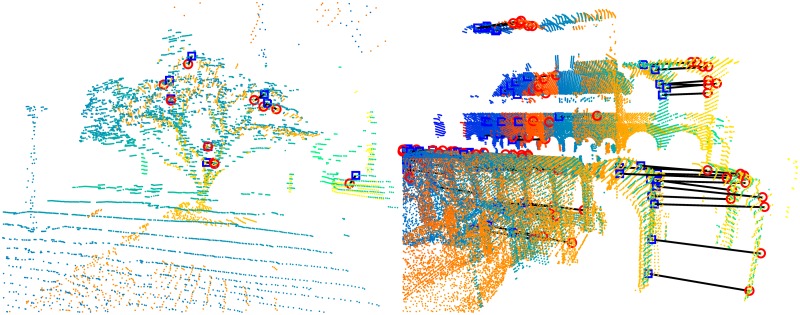
Consensual feature correspondences. The reading and reference point clouds are displayed in orange and blue tones, respectively, aligned with each other using the ground-truth pose. Black lines connect the corresponding features from the consensual set, i.e., the inliers, marked by red circles and blue squares. The markers would be concentric in case of a perfect match. (left) Accurate pose estimate from 19 inliers in Gazebo point clouds with overlap 0.5. (right) Inaccurate translation estimate from 99 inliers in ETH point clouds with overlap 0.67.

Interesting fail cases are obtained with the *ETH* dataset—despite all methods failing to provide a reasonable translation estimate in most cases (A50 ≥ 2.37m), the feature-based methods consistently provide very accurate estimates of rotation. Moreover, our method even achieves the highest rotation accuracy on this dataset, contrary to a rather low translation accuracy. This is due to the regular structure of the environment with many repetitive patterns with similar orientation—even if these features are mismatched with each other, the rotation can still be estimated correctly from their correspondences. See Fig 6 for a visualization of consensual feature correspondences.

Average registration errors across all datasets are summarized in Table 2, together with running times. The proposed method provides the most accurate estimates of rotation for all reading-reference pairs with hard initial poses, while 3D-NDT provides the most accurate estimates of translation. For relative overlap at least 75 percent, nevertheless, the proposed method provides superior accuracy in both rotation and translation. Average running time of our method, 14s, is less than 6× higher than that of the fastest local method, which is the point-to-point ICP, and about 3× lower than that of the second fastest global method, which is K-4PCS. Having been implemented in Matlab, our method can still benefit from further optimizations achieved by using a compiled language. Other methods were implemented in C++ as a part of the Point Cloud Library [21].

**Table 2:** Average errors and running times of registration methods.

Method	Rotation	Translation	Time
Point [1, 4]	0.97 (0.85)	2.60 (1.52)	**2.5** ± 2.0 s
Plane [2, 4]	1.05 (0.91)	3.09 (1.89)	4.1 ± 2.9 s
G-ICP [3]	0.83 (0.71)	2.71 (1.80)	3.1 ± 2.9 s
3D-NDT [9]	0.84 (0.70)	**1.98** (1.29)	4.7 ± 3.4 s
FPFH [5]	0.59 (0.19)	3.13 (1.00)	119.5 ± 80.1 s
4PCS [10]	0.85 (0.45)	3.57 (1.67)	47.0 ± 272.8 s
K-4PCS [11]	1.21 (0.87)	4.67 (2.80)	55.8 ± 154.3 s
Ours	**0.32** (**0.03**)	2.66 (**0.58**)	14.0 ± 18.8 s

All errors listed are on *hard* poses, rotation error *e*_r_ is in radians, translation error *e*_t_ in meters, time shown is mean ± standard deviation. Errors for point cloud pairs of relative overlap at least 75 percent are listed in brackets. The best result is printed in bold. All methods but ours are implemented in C++ as part of the Point Cloud Library [21], ours is implemented in Matlab.

## Conclusion

In this paper, we extended the local features of [15] by introducing keypoint detection and using a more robust method of the underlying local reference frames. The method was evaluated on a set of challenging real-world datasets [12]. We compared the method to state of the art—two local-optimization-based methods (Generalized ICP [3] and 3D Normal-Distribution Transform [9]) and three global-search-based methods (Fast Point-Feature Histograms with SAC [5] and two variants of the 4-Points Congruent Set method [10, 11]). The experimental protocol from [4] provided a sufficient level of difficulty for both classes.

Failures of the feature-based methods, ours and [5], are mostly due to low overlap between the point clouds and repetitive structures which prevail in some of the scenes, especially in the *ETH* dataset. Nevertheless, for overlap ratios above 0.75 the proposed method achieves the highest accuracy and could also be used to initialize the local methods which achieves high accuracy when an initial estimate within a basin of convergence is provided.

The evaluation of its underlying components suggests that the points constitute a more solid base for both detecting keypoints and establishing local reference frames than the surface normals. Local maxima of the smallest eigenvalue of the feature covariance matrix provide most repeatable keypoints for both of the feature types. Note that this corresponds to the well-known method image-based keypoint detector of [22].

Sign disambiguation of the basis vectors proved to be a very important aspect in creating repeatable local reference frames. For situations in which the sensor position is not known, we proposed a novel method which achieves better repeatability than the general method of [16] used in the SHOT descriptor [17]. The results also confirmed that the sensor position, when it is known, provides a very informative clue for sign disambiguation and justified its usage therein. Another conclusion can be made regarding which vectors should ensure a right-handed coordinate system—vectors close to surface normal are the easiest to disambiguate and should thus be used preferably.

We see many possibilities for improving the overall accuracy of registration which can be addressed in future work, namely

- introducing a verification step to ensure that the geometric constraints are met and the open-space assumption is not violated,
- detecting repetitive structures to reduce mismatched features, or
- using higher-level knowledge to identify, recognize, and match distinguished objects in the scene.
